# Remembering Components of Food in *Drosophila*

**DOI:** 10.3389/fnint.2016.00004

**Published:** 2016-02-19

**Authors:** Gaurav Das, Suewei Lin, Scott Waddell

**Affiliations:** Centre for Neural Circuits and Behaviour, University of OxfordOxford, UK

**Keywords:** insects, *Drosophila*, dopamine, neural circuits, food, memories, internal states

## Abstract

Remembering features of past feeding experience can refine foraging and food choice. Insects can learn to associate sensory cues with components of food, such as sugars, amino acids, water, salt, alcohol, toxins and pathogens. In the fruit fly *Drosophila* some food components activate unique subsets of dopaminergic neurons (DANs) that innervate distinct functional zones on the mushroom bodies (MBs). This architecture suggests that the overall dopaminergic neuron population could provide a potential cellular substrate through which the fly might learn to value a variety of food components. In addition, such an arrangement predicts that individual component memories reside in unique locations. DANs are also critical for food memory consolidation and deprivation-state dependent motivational control of the expression of food-relevant memories. Here, we review our current knowledge of how nutrient-specific memories are formed, consolidated and specifically retrieved in insects, with a particular emphasis on *Drosophila*.

## Introduction

All foraging animals have to obtain an optimal balance of nutrients from a variety of available food sources. In addition, nutrient demands change as animals age, reproduce, migrate, face predators and overcome immune challenges. Animals must therefore constantly adjust their foraging strategies to meet these nutritional needs. Remembering details of prior successful feeding experience can aid foraging so that the useful food sources can be found and appropriate nutrients consumed when required. Avoiding toxic and potentially harmful sources is also important. Insects can learn to associate food-related smells, tastes, colors and textures as predictors of potentially nutritious or harmful food (Papaj and Prokopy, 1989; Dukas, 2008; Hollis and Guillette, 2011). Learning improves the efficiency of foraging and evolutionary fitness (Dukas and Bernays, 2000; Dukas and Duan, 2000; Raine and Chittka, 2008). It therefore seems likely that natural selection has honed mechanisms that produce efficient foraging strategies.

In *Drosophila* the neurobiology of food and water-reinforced memory can be studied using simple associative learning paradigms where groups of hungry or thirsty flies associate an odor with consumption of food or water (Tempel et al., 1983; Krashes and Waddell, 2008; Colomb et al., 2009; Lin et al., 2014). Hunger and thirst preferentially promote efficient expression of either the sugar or water memories. These assays combined with genetic control in *Drosophila* permit an investigation of neural mechanisms through which learning influences efficient foraging behavior. In this review, we provide examples of food-driven behavior from a variety of insects, but mostly focus on recent studies in *Drosophila.* Work in the fruit fly supports a provocative model that the anatomical segregation of dopaminergic neurons (DANs) might provide a neural substrate across which specific food component memories can be formed and deprivation state-dependent memory expression might be controlled.

## Dopaminergic Neurons Reinforce Food Component Memories

**Figure 1:**
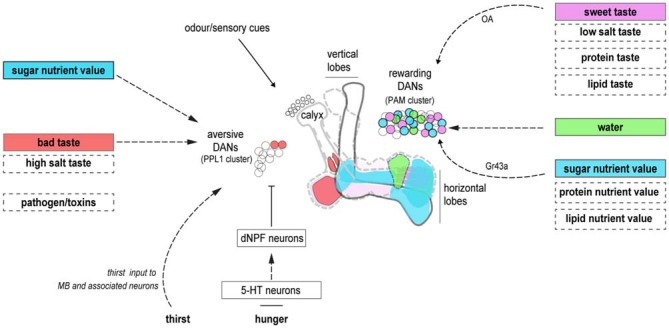
**Dopaminergic neurons (DANs) innervating the mushroom bodies (MBs) provide reinforcement and motivational control.** DANs innervating unique zones of the MB lobes may represent the reinforcing properties of individual food components. Reinforcement from the sweet taste and energetic value of sugars are segregated in rewarding DANs. A unique water reinforcement zone may exist on the horizontal MB lobe. The taste and energetic elements of other nutrients may involve different subsets of DANs. Aversive DANs provide the reinforcing properties of bad taste. They also control nutrient-dependent consolidation, and hunger-dependent expression, of carbohydrate memories. It is currently unclear if similar processes represent other deprivation states. Dashed boxes denote food components and dashed arrows, neuronal pathways that remain be delineated in fruit flies. Cell body colors correspond to their relevant innervation zones on the MB. The diagram is not intended to be anatomically accurate.

Insect mushroom bodies (MBs) are large ensembles of parallel projecting neurons (ranging from approximately 500,000 in the honeybee to 5000 in fruit flies) that appear to function as a multimodal association network in which memories are formed and stored, and behaviors are controlled (Vowles, 1964; Menzel et al., 1974; Heisenberg, 1980; Heisenberg et al., 1985; de Belle and Heisenberg, 1994; Mizunami et al., 1998; Strausfeld et al., 1998; Ikeda et al., 2005; Vogt et al., 2014; Kirkhart and Scott, 2015; Figure 1). Individual MB neurons, or Kenyon cells (KCs), receive input in the calyx and surrounding areas from olfactory, visual, gustatory and tactile streams from the periphery (Ito et al., 1998; Strausfeld et al., 1998). Individual odors are represented as activity in relatively sparse subsets of the overall population of KCs (Perez-Orive et al., 2002; Ito et al., 2008; Honegger et al., 2011).

Dopaminergic neurons (DANs) innervating the MB are critical for learning the value of beneficial and harmful food components. Anatomically discrete DANs provide valence-specific learning signals to different regions on the mushroom body lobes (Riemensperger et al., 2005; Claridge-Chang et al., 2009; Aso et al., 2012, 2014a; Burke et al., 2012; Liu et al., 2012; Waddell, 2013; Figure 1). There, dopamine release is believed to modify output synapses of coincident odor-activated KCs (Heisenberg, 2003; Owald and Waddell, 2015). This organization, taken with the large number and anatomical diversity of rewarding DANs, is supportive of a general model that nutrient-specific associative memories may be formed within different MB zones that are innervated by the relevant DANs (Aso et al., 2014a; Lin et al., 2014; Huetteroth et al., 2015; Yamagata et al., 2015). With this model in mind, we will discuss the fields’ current knowledge of learning with specific food components.

### Carbohydrate Learning

Carbohydrates are an essential source of energy and many insects including bees, ants, cockroaches, crickets and fruit flies can be trained to associate sensory cues with sugar consumption (Kuwabara, 1957; Takeda, 1961; Nelson, 1971; Fukushi, 1973; McGuire and Hirsch, 1977; Bitterman et al., 1983; Tempel et al., 1983; Yuval and Galun, 1987; Sakura and Mizunami, 2001; Scherer et al., 2003; Neuser et al., 2005; Gerber and Stocker, 2007; Krashes and Waddell, 2008; Schipanski et al., 2008; Colomb et al., 2009; Josens et al., 2009; Menzel, 2012; Rohwedder et al., 2012; Apostolopoulou et al., 2013). Following a 2 min pairing of odor and sucrose, adult *Drosophila* form memories that can be immediately expressed and last for days (Krashes and Waddell, 2008; Colomb et al., 2009). Both the sweet taste and nutrient value of a sugar contribute to memory reinforcement (Burke and Waddell, 2011; Fujita and Tanimura, 2011). Training with arabinose or xylose, sugars that taste sweet but that fruit flies cannot metabolize, only forms short-term memory. However, if arabinose or xylose is supplemented with nutritious but tasteless sorbitol flies form long-term memory (Burke and Waddell, 2011).

The segregation of sweet taste and nutrient value reinforcement is evident at the level of the DANs (Figure 1). Two distinct subsets of DANs convey sweet taste and nutrient value reinforcement (Huetteroth et al., 2015; Yamagata et al., 2015). Sweet-taste DANs are activated by octopamine through the OAMB α-adrenergic like receptor (Burke et al., 2012; Huetteroth et al., 2015). Consequently, flies that cannot synthesize or release octopamine are unable to reinforce short-term memories but they show normal long-term memory when conditioned with sucrose (Schwaerzel et al., 2003; Das et al., 2014). Furthermore, pairing odor presentation with artificial activation of the OAMB-expressing DANs implants only a short-term memory (Huetteroth et al., 2015; Yamagata et al., 2015). A similar pairing of odor-presentation with artificial activation of octopaminergic neurons forms an appetitive short-term memory in larvae and adult flies (Schroll et al., 2006; Burke et al., 2012).

In the honeybee the octopaminergic VUMmx1 neuron responds to sucrose and electrical stimulation of VUMmx1 can substitute for sugar-reward in olfactory learning (Hammer, 1993). It seems possible that VUMmx1-released octopamine might also provide sweet-taste reinforcement via subsets of DANs in the bee.

Blocking nutrient value DANs during sucrose learning specifically impairs long-term memory (Huetteroth et al., 2015; Yamagata et al., 2015). In addition, pairing direct activation of the nutrient value DANs with odor can implant a long-term memory. Although nutrient information is available to direct behavior minutes after training, work suggests that a delayed post-ingestive signal is also required to drive long-term memory consolidation (Burke and Waddell, 2011). Pharmacological block of the intestinal glucose transporter specifically impairs D-glucose reinforced LTM (Musso et al., 2015). Interestingly, the activity of aversive DANs that were known to signal satiety (Krashes et al., 2009) is increased following ingestion of nutritious sugar, and forcing their activity after training can facilitate long-term memory (Musso et al., 2015). Memory consolidation also requires activity after training in a plausible recurrent network loop from MB neurons to glutamatergic MB output neurons (MBONs) to rewarding DANs. Blocking any of the contributing neurons after training impaired long-term sugar-reinforced memory (Ichinose et al., 2015). Taken together these experiments suggest ongoing activity in a distributed set of DANs may provide post-ingestive nutrient value information to reinforce long-term memory. It will be important to establish how the two reported mechanisms relate and whether they are triggered together. Neurons in the brain expressing the GR43a fructose receptor have been suggested to provide nutrient value input to DANs (Miyamoto et al., 2012; Yamagata et al., 2015).

### Amino Acid Learning

Amino acids are essential building blocks of proteins for growth, development and reproduction. For some insects, such as tsetse flies, Colorado beetles and blowflies, amino acids can provide energy to fuel flight (Bursell, 1963; Sacktor and Childress, 1967; de Kort et al., 1973). Proteins and specific amino acids are also critical in the diet for egg production and fertility in blowflies, and other species of fruit flies including *Drosophila* (Grandison et al., 2009; Harwood et al., 2013).

Ample evidence suggests that amino acids are discretely valued from sugars in the insect brain. Honeybees and butterflies show a preference for nectars that contain certain amino acids (Inouye and Waller, 1984; Hendriksma et al., 2014). Female blowflies consume more proteins than males after eclosion and furthermore, mated females show peaks of protein consumption following each bout of egg production (Strangways-Dixon, 1959, 1961; Dethier, 1961, 1976). Mated female *Drosophila* shows a similar preference for protein-rich food over sugar, compared to males and virgin females, after a period of protein deprivation (Ribeiro and Dickson, 2010; Vargas et al., 2010). However this switch in preference after mating is independent of egg production and is mediated instead by sex peptide, which is transferred with the male seminal fluid to the female during copulation (Ribeiro and Dickson, 2010). Adult fruit flies also prefer to eat amino acids rather than glucose when protein-deprived (Toshima and Tanimura, 2012). Work in *Drosophila* larvae suggests that DANs are involved in amino acid evaluation. Larvae avoid eating food that lacks essential amino acids and this behavior requires the GCN2 amino acid sensor in three larval DANs (Bjordal et al., 2014).

Amino acids can also reinforce learning. Locusts and cockroaches can be trained to associate odors or colors with protein-rich food (Raubenheimer and Tucker, 1997; Gadd and Raubenheimer, 2000) and honeybees can be trained with odors reinforced with sugar containing an amino acid (Simcock et al., 2014). Although *Drosophila* larvae can be reward-conditioned using non-essential aspartic acid (Schleyer et al., 2015) such a phenomenon remains to be demonstrated in adult flies.

### Water Learning

Water is essential for cell function and is perhaps the most critical nutrient for a small insect that can easily desiccate. Water-deprived insects, show robust approach behavior to water vapor and water-associated sensory cues (Raubenheimer and Blackshaw, 1994; Matsumoto and Mizunami, 2002a; Unoki et al., 2006; Lin et al., 2014). In adult *Drosophila*, learning to associate an odor with drinking water requires the action of DANs that are different to those that are required for reinforcement with nutritious sugar (Lin et al., 2014). At present the water DANs have not been functionally segregated from those that reinforce the sweet taste of sugar. However, water learning does not require octopamine suggesting that water and sweet-taste DANs may also innervate separate zones on the MB lobes. Water-associated memory persists for 6–10 weeks in the cricket (Matsumoto and Mizunami, 2002b). In *Drosophila* perdurance of water memory appears to correlate with the amount of water ingested during training. Drinking more leads to a longer lasting memory and under certain conditions 24 h water memory was observed (Lin et al., 2014).

### Bad Tastes and Toxins

Avoiding consumption of harmful food also provides an obvious survival advantage. Many toxins are repellents by virtue of their bad taste, which allows insects to reject toxin-laden food using multiple layers of taste detection (recently reviewed in Freeman and Dahanukar, 2015; Joseph and Carlson, 2015). In addition, honeybees can learn to avoid odors or visual stimuli associated with toxic compounds that they can taste (Wright et al., 2010; Wright, 2011). Learned bitter taste aversion in the honeybee requires dopamine (Wright et al., 2010).

*Drosophila* larvae can be conditioned to associate an odor with aversive bitter-tasting quinine (Gerber and Hendel, 2006; Schleyer et al., 2011; Apostolopoulou et al., 2014). Hungry adult flies can be coaxed to consume bitter-tasting compounds if they are mixed with a high enough concentration of sugar (Das et al., 2014). Flies trained with a bitter-sugar mixture show immediate avoidance of the conditioned odor, which later switches to conditioned odor approach. This suggests the flies form a labile aversive memory and a lasting approach memory that compete to guide behavior (Das et al., 2014). The bitter-sugar mixture activates the aversive and rewarding DANs together (Das et al., 2014; Harris et al., 2015). Although it is not known whether all aversive compounds engage the same DANs, those activated by bitter-taste are also required for aversive learning with electric shock and high heat (Schwaerzel et al., 2003; Claridge-Chang et al., 2009; Aso et al., 2010, 2012; Galili et al., 2014). Therefore the aversive DANs may only code the magnitude of an aversive stimulus and not its quality (Das et al., 2014; Galili et al., 2014).

Grasshoppers, desert locusts and honeybees can also learn the post-ingestive consequences of consuming toxic food (Behmer et al., 1999; Wright et al., 2010; Simoes et al., 2012). In honeybees memory of post-ingestive malaise develops over time and requires serotonin, 5-HT (Wright, 2011). Adult *Drosophila* can also learn to avoid an odor that was associated with pathogen-tainted food (Babin et al., 2014). Since the flies could not taste the intestinal pathogen in this study, it is possible that the learned aversion is reinforced by post-ingestive malaise (Hurst et al., 2014).

### Salt Learning

Salt is essential for osmotic balance and many physiological processes and insects actively regulate their salt intake (Trumper and Simpson, 1993; Simpson et al., 2006; Simpson and Raubenheimer, 2012). Mated female *Drosophila* exhibit an enhanced gustatory response for salt and increase salt consumption. This increased salt appetite, like the learning-independent change in protein preference (Ribeiro and Dickson, 2010), is driven by male sex peptide transferred to the female during copulation (Walker et al., 2015).

Insects such as locusts and crickets can learn to associate specific sensory cues with salt or salt infused food. Interestingly, whereas locusts were shown to approach the salty food associated cue, crickets showed learned avoidance of a salt reinforced cue (Trumper and Simpson, 1994; Unoki et al., 2006). Studies of salt learning in larval *Drosophila* may provide an explanation for this apparent conundrum. Larvae are attracted to odors paired with low salt concentrations but avoid odors previously paired with higher salt concentrations (Niewalda et al., 2008; Russell et al., 2011). Assuming adult fruit flies can be conditioned with salt, one might predict that high salt learning would activate aversive DANs while lower concentrations might preferentially recruit rewarding DANs. This could simply reflect the different gustatory neurons that are activated by low and high salt concentrations (Hiroi et al., 2004; Zhang et al., 2013).

### Alcohol Learning

Insects encounter low levels of ethanol in rotting fruits and it has been reported that consuming ethanol enhances fitness of larvae and adult fruit flies (Geer et al., 1993; Bokor and Pecsenye, 2000; Devineni and Heberlein, 2013). Female fruit flies also have a preference for laying eggs on ethanol-containing food (Azanchi et al., 2013).

Adult fruit flies can also be conditioned with odors reinforced with ethanol vapor. Their performance after training shows a similar profile to flies conditioned with bitter-tainted sugar (Das et al., 2014); early aversion later switches to approach (Kaun et al., 2011) consistent with a model that alcohol also reinforces parallel appetitive and aversive memories (Aso et al., 2014b). Surprisingly, broad manipulation of DANs suggested that they are dispensable for alcohol to reinforce learned aversion and approach, but are required for expression of longer-term alcohol-conditioned approach (Kaun et al., 2011). It therefore remains unclear whether specific DANs contribute to alcohol reinforcement.

## Specific Deficit Promotes Appropriate Memory Expression

Efficient foraging requires insects to utilize their learned behaviors at the appropriate time. Studies of locusts and cockroaches suggest that insects possess a sophisticated level of control that permits nutrient-specific deficits to select the relevant procurement behaviors. Following training to associate colors or odors paired with synthetic foods that are either rich in carbohydrate or protein, they chose the cue predicting carbohydrate if sugar deprived, but the cue predicting protein if protein deprived (Raubenheimer and Tucker, 1997; Gadd and Raubenheimer, 2000). Work in the fruit fly again suggests possible mechanisms to accomplish this level of nutrient-deficit dependent control based on reward expectation.

Sugar-conditioned *Drosophila* most efficiently approach the previously rewarded odor only when they are hungry (Krashes and Waddell, 2008; Krashes et al., 2009). The state of hunger is broadcast throughout the brain by multiple monoamine and neuropeptide signals to control feeding, energy expenditure, the gain of sensory neurons, nutritional homeostasis and sugar memory expression (reviewed in Audsley and Weaver, 2009; Nässel and Winther, 2010; Pool and Scott, 2014). *Drosophila* Neuropeptide F (dNPF), an orthologue of mammalian neuropeptide Y (Brown et al., 1999) mediates hunger-dependent control of sugar memory expression by modulating the activity of a subset of aversive DANs that innervate the MB (Krashes et al., 2009; Figure 1). A model suggests that in the food-satiated state, the tonic activity of aversive DANs on the MB inhibits the expression of sugar memory. When flies are starved, dNPF release inhibits the aversive DANs, releasing sugar memory expression (Krashes et al., 2009).

Artificial activation of a subset of 5-HT expressing neurons in satiated flies also releases sugar memory expression in addition to promoting general feeding behaviors (Albin et al., 2015). Therefore 5-HT neurons may be upstream of dNPF neurons in signaling nutritional status mediating motivational control of sugar memory expression.

Expression of sugar memory can also be suppressed by ingestion of a high osmolarity nutritious or non-nutritious solution (Gruber et al., 2013). This reported lack of nutrient-specificity seems somewhat counter-intuitive and the adaptive relevance of such a non-specific suppression of food-related memory expression is currently unclear.

Importantly, thirst and hunger states provide independent control over memory expression. Whereas thirsty flies most efficiently express water memory, hungry flies preferentially express sugar memory (Lin et al., 2014). It therefore seems possible that the expression of other nutrient-specific memories will be controlled by independent, perhaps DAN-dependent, neural mechanisms in the fly.

Interesting work with salt in rats provides a more extreme example of how predictive evaluation can be robustly changed by internal nutrient deficit. Rats taught to avoid a metal lever paired with high aversive concentrations of salt, avidly approach the same lever when they are deprived of sodium (Robinson and Berridge, 2013). Establishing a similar paradigm in *Drosophila* could be informative.

## Conclusion

In summary, work suggests that foraging insects learn about multiple components of their food. Subsequently their behavior can be directed by their knowledge towards a specific goal of neutralizing a particular nutrient deficit. We propose that nutrient components might be differentially represented in subsets of reinforcing DANs so that carbohydrate, protein, lipid (Toshima and Tanimura, 2012; Masek and Keene, 2013), water and salt memories can be independently coded (Figure 1). In addition, other combinations of DANs might promote the expression of these nutrient-specific memories by increasing the valuation of the predictive cues, through gating of the relevant parts of the MBON network (Owald and Waddell, 2015). Rigorously testing these models in *Drosophila* may uncover general organizational principles of how a dopaminergic evaluation system operates.

## Author Contributions

The article was written with the input of all three authors.

## Funding

SL was supported by an EMBO Advanced Fellowship. SW is funded by a Wellcome Trust Senior Research Fellowship in the Basic Biomedical Sciences and by funds from the Gatsby Charitable Foundation, the Oxford Martin School and the Bettencourt-Schueller Foundation.

## Conflict of Interest Statement

The authors declare that the research was conducted in the absence of any commercial or financial relationships that could be construed as a potential conflict of interest.
